# EMG-YOLO: road crack detection algorithm for edge computing devices

**DOI:** 10.3389/fnbot.2024.1423738

**Published:** 2024-07-02

**Authors:** Yan Xing, Xu Han, Xiaodong Pan, Dong An, Weidong Liu, Yuanshen Bai

**Affiliations:** ^1^School of Transportation and Surveying Engineering, Shenyang Jianzhu University, Shenyang, Liaoning, China; ^2^Shenyang Boyan Intelligent Transportation Technology Co., Ltd., Shenyang, Liaoning, China; ^3^Shenyang Public Security Bureau Traffic Police Division, Shenyang, Liaoning, China

**Keywords:** road crack detection, YOLOv5, Efficient Decoupled Head, MPDIou, deep learning

## Abstract

**Introduction:**

Road cracks significantly shorten the service life of roads. Manual detection methods are inefficient and costly. The YOLOv5 model has made some progress in road crack detection. However, issues arise when deployed on edge computing devices. The main problem is that edge computing devices are directly connected to sensors. This results in the collection of noisy, poor-quality data. This problem adds computational burden to the model, potentially impacting its accuracy. To address these issues, this paper proposes a novel road crack detection algorithm named EMG-YOLO.

**Methods:**

First, an Efficient Decoupled Header is introduced in YOLOv5 to optimize the head structure. This approach separates the classification task from the localization task. Each task can then focus on learning its most relevant features. This significantly reduces the model’s computational resources and time. It also achieves faster convergence rates. Second, the IOU loss function in the model is upgraded to the MPDIOU loss function. This function works by minimizing the top-left and bottom-right point distances between the predicted bounding box and the actual labeled bounding box. The MPDIOU loss function addresses the complex computation and high computational burden of the current YOLOv5 model. Finally, the GCC3 module replaces the traditional convolution. It performs global context modeling with the input feature map to obtain global context information. This enhances the model’s detection capabilities on edge computing devices.

**Results:**

Experimental results show that the improved model has better performance in all parameter indicators compared to current mainstream algorithms. The EMG-YOLO model improves the accuracy of the YOLOv5 model by 2.7%. The mAP (0.5) and mAP (0.9) are improved by 2.9% and 0.9%, respectively. The new algorithm also outperforms the YOLOv5 model in complex environments on edge computing devices.

**Discussion:**

The EMG-YOLO algorithm proposed in this paper effectively addresses the issues of poor data quality and high computational burden on edge computing devices. This is achieved through optimizing the model head structure, upgrading the loss function, and introducing global context modeling. Experimental results demonstrate significant improvements in both accuracy and efficiency, especially in complex environments. Future research can further optimize this algorithm and explore more lightweight and efficient object detection models for edge computing devices.

## 1 Introduction

The cyclical effects of vehicle loads and the long-term erosion of natural environmental factors combine to affect the road structure. There is a significant decline in the service function of the road in the middle and later stages of its use. Not only does road degradation threaten the safety of motorists, it can also cause congestion in traffic flow and shorten the overall life of the road infrastructure. As a result, road crack detection has become an important means of extending the life of roads. However, in the actual road crack detection project, the complexity of the road environment makes it difficult for automated detection equipment to meet the needs of the actual project in terms of recognition accuracy. Thus, the accuracy of road crack detection algorithms needs to be further improved.

Object detection algorithms show significant advances in the field of road crack detection. Among them, semantic segmentation (Ronneberger et al., 2015; Liu et al., 2021; Xu et al., 2021; Zhang W. et al., 2021; Tan et al., 2022) enables accurate labeling of crack regions down to the pixel level. This algorithm allows the fine-grained capture and differentiation of morphological features of pavement cracks. Nonetheless, the high annotation cost barrier of semantic segmentation becomes a major constraint to its widespread popularity. With the continuous breakthrough of deep learning technology (Maeda et al., 2018; Nhat-Duc et al., 2018; Hou et al., 2022; al-Huda et al., 2023; Talaei et al., 2023), it brings new opportunities for road crack detection. The mainstream object detection models are Faster R-CNN (Ren et al., 2016), SSD (Liu et al., 2016), EfficientDet (Tan et al., 2020), and CenterNet (Resnet50; Duan et al., 2019). Whereas Faster R-CNN, due to its fine region cropping and subsequent refined classification and regression steps. The algorithm performs better in terms of accuracy. SSDs are more suitable for speed sensitive application scenarios. EfficientDet, on the other hand, combines the advantages of both, ensuring higher detection accuracy while improving operational efficiency and model scalability. CenterNet (Resnet50) converges more easily during training and can achieve better results for training with finite resources. With the continuous iterative updating of the YOLO algorithm (Redmon et al., 2016), the model of the YOLOv5 framework has become one of the mainstream solutions in the field. In response to the challenges of complex and diverse scenarios in road crack detection, researchers have proposed various improvements to enhance the accuracy of the model. Ren et al. (2023) proposed a model named YOLOv5s-M based on YOLOv5 which is capable of handling large-scale detection layers. The algorithm improves the detection accuracy of urban road crack objects. However, the model handles large-scale detection layers, which may increase the computational complexity and affect real-time performance. Tang et al. (2024) proposed a crack detection algorithm based on improved YOLOv5s for asphalt pavement crack detection under complex pavement conditions (affected by glare, road surface water, debris, etc.) with low recognition accuracy. The results show that the improved YOLOv5s model has better detection accuracy under complex pavement conditions. While the model performs well under complex pavement conditions, the model may have been over-fitted to specific environmental conditions with limited generalization. Guo and Zhang (2022) proposed the MN-YOLOv5 pavement damage detection algorithm. Algorithm uses a new backbone feature extraction network and attention module. Size of the model is reduced by about 1.62 times. The accuracy is improved by 2.5 percent. However, the experimental results may lack diverse test data and do not fully demonstrate the performance of the model in different scenarios. Aghayan-Mashhady and Amirkhani (2024) developed an algorithm for detecting road damage based on YOLOv5 with several different baseline models. The algorithm utilizes traditional bounding box enhancement and road damage generation adversarial network based enhancement techniques. New models improve the accuracy of road damage detectors in different environments and field conditions. However, the introduction of GAN may increase the complexity and computational cost of the model and affect the real-time detection performance. Dai et al. (2021) proposed the Dyhead dynamic object detection head. Multiple self-attention mechanisms are coherently combined between feature layers for scale-awareness, between spatial locations for spatial-awareness, and within the output channel for task-awareness. This method significantly improves the detection accuracy of the YOLOv5 object detection head without adding any computational overhead. While the Dyhead dynamic target detection head improves detection accuracy, the combination of multiple self-attention mechanisms may increase the computational overhead and affect the real-time performance of the model. Qiao et al. (2021) proposed Switchable Atrous Convolution (SAconv). It convolves features at different Atrous rates and collects the results using switching functions. SAconv combines them to form DetectoRS, which greatly improves the accuracy of YOLOv5. However, the method may be effective in specific scenarios, but the ability to generalize to other scenarios needs further validation. Wei et al. (2023) proposed a YOLOv5s-BSS to address the limitations of existing state-of-the-art crack detection methods in terms of accuracy and detection speed. The algorithm was compared to YOLOv5s on road damage datasets from China, Japan, and the USA with higher crack detection accuracy. However, the introduction of modules such as BiFPN and SPPCSPC may increase the model complexity and affect the real-time performance. Jiang et al. (2023) proposed an RDD-YOLOv5 to address the complexity of the road crack background, low resolution and high similarity of cracks. The model’s ability to accurately identify road cracks and the average accuracy are better than the original YOLOv5, with an average accuracy of 91.48%, which is 2.5% better than the original YOLOv5. However, the experimental results are mainly based on specific datasets and lack validation against more diverse scenarios and data. Hu et al. (2024) proposed an automated 3D crack detection system for structures based on high-precision Light Detection Ranging (LiDAR) and camera fusion. Through the extraction of high-precision 3D crack features, the significant measurement accuracy reaches sub-millimeter level (0.1 mm) when compared with the measurement results of traditional methods. However, the dependence on LiDAR equipment limits the practical application of the method, especially in resource-constrained situations.

Although YOLOv5-based algorithms have made significant progress in road crack detection accuracy. However, current research has not yet fully explored the effective integration of the improved YOLOv5 model with edge computing devices. Edge devices, due to their inherent miniaturization, usually carry limited processor power, memory size and storage space. This creates a stark hardware configuration gap compared to centralized high-performance computers or servers. Such devices are often difficult to support YOLO while meeting the requirements of low power consumption and compact size. However, it is often difficult to support the massive floating-point operations required during the implementation of the YOLOv5 model. This leads to a decrease in model detection accuracy. Therefore, how to maintain or even optimize the detection accuracy on the premise of achieving YOLOv5 model for edge computing architecture is highly adaptable and efficient operation. This has become a key technology and challenge to be solved.

Based on the above, Liang et al. (2022) proposed an object detection (OD) system based on edge cloud cooperation and reconfigured convolutional neural networks, called edge YOLO. The system can effectively avoid over-reliance on computing power and uneven distribution of cloud computing resources. The model can maximize the efficiency of multi-scale prediction. However, the model is a lightweight OD framework implemented by combining pruned feature extraction network and compressed feature fusion network. The pruning operation removes weights or channels that are considered unimportant in the network. This may lead to information loss, which in turn affects the detection accuracy of the model. Ganesh et al. (2022) proposed a new edge GPU friendly multi-scale feature interaction module. The algorithm utilizes the existing state-of-the-art methods in the lost combinatorial connections between various feature scales. This can improve the accuracy and execution speed of various edge GPU devices available in the market. However, the algorithm uses the older YOLO v4 model and is not adapted to the latest models. Li et al. (2021) designed an edge to client road damage detection system based on YOLO object detection algorithm. The system includes roadside information acquisition platform, edge computing device, cloud transmission system and client. The experimental results show that the system can achieve real-time display of road damage detection. However, the system does not solve the accuracy degradation of YOLOv5 in the edge computing device due to the poor quality data collected. Zhang Y. M. et al. (2021) proposed a lightweight detector, CSL-YOLO. The model was modeled by proposing a new lightweight convolutional method cross-level lightweight (CSL) module. The CSL module is used to generate redundant features from cheap operations and the proposed CSL-Module can significantly reduce the computational cost. However, the model is not optimized for the specific problem of road crack detection and its performance in road crack detection is not very satisfactory.

To address the problem of accuracy degradation caused by poor quality data collected due to the complexity of the real environment in edge computing devices. In this paper, an improved YOLOv5 object detection model, EMG-YOLO, is proposed. The model performance is strengthened through the introduction of Efficient Decoupled Head (EDH) by decoupling mechanism. The optimization of the IOU loss function, as well as the improvement of the C3 module and the Head part of the model, successfully enhanced the overall performance of the model. The successful application of the method in road crack detection verifies the feasibility of the method. The main contributions of this paper are as follows:.

1. The Efficient Decoupled Head addresses the issue of information confusion and task conflict arising from the shared feature map for classification and regression tasks in the traditional YOLOv5 model, thereby enhancing overall performance.
2. The shapes of road cracks vary greatly, and traditional IoU performs poorly in handling elongated or irregularly shaped cracks. The MPDIOU function better adapts to various crack shapes by upgrading the conventional IoU function, resolving the issue of inaccurately reflecting prediction accuracy in cases where bounding boxes are highly overlapping but differ in shape.
3. To tackle the problem of target features being easily obscured by background noise during detection on edge devices, the Global Context Block is introduced to optimize the C3 module, thereby improving the feature representation capability of the YOLOv5 model.

## 2 YOLOv5 network architecture

**Figure 1:**
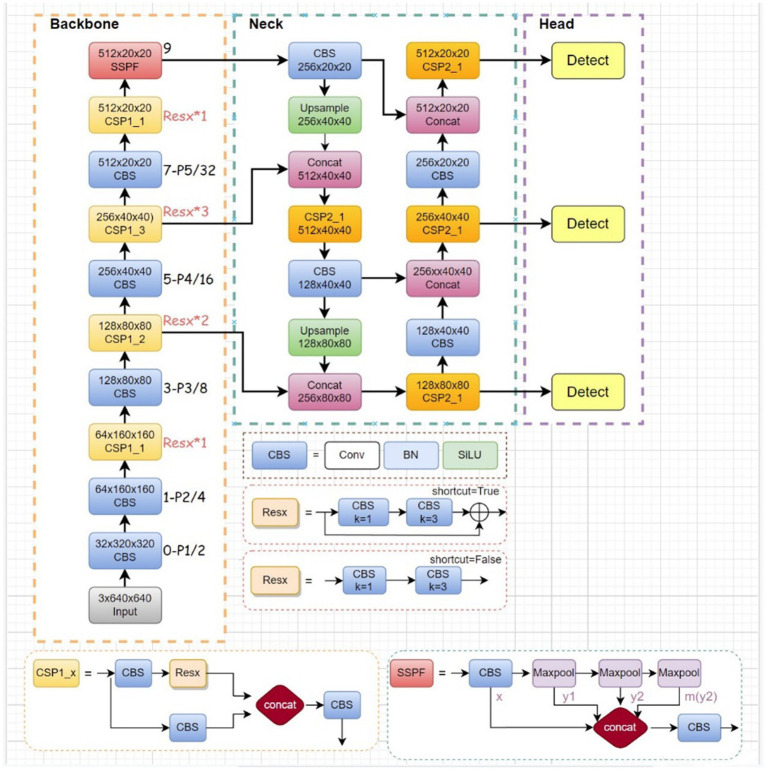
Structure of YOLOv5.

When compared with the traditional two-stage detector, YOLOv5 exhibits superior detection speed and enhanced accuracy. Its network architecture comprises three essential components: Backbone, Neck, and Head. Moreover, the YOLOv5 algorithm has been fine-tuned for parameter count and inference speed optimizations in contrast to YOLOv4. Figure 1 illustrates the structure of YOLOv5.

Implementing YOLOv5 on edge computing devices presents the challenge of varying data quality. This not only heightens the model’s difficulty in handling noisy data but also risks excessive consumption of computational resources. Thereby impacting model recognition accuracy. To address this, a series of optimizations were implemented on the YOLOv5 model. Firstly, an efficient decoupled head structure was introduced to expedite the model’s training convergence speed. Secondly, fine tuning of the loss function and adoption of the MPDIOU loss function were carried out to alleviate computational burdens during training. Finally, the traditional convolutional layer was replaced with the GCC3 module to reduce both computational complexity and parameter count.

## 3 Enhancement of road defect detection network architecture based on the YOLOv5 model

### 3.1 Constructing a hybrid channel strategy for the detection head

The demanding computational resources and lengthy training time required during the model training phase contribute to this issue. In constructing deep learning models using the YOLOv5 framework, minimizing the required iterations is pivotal to enhancing model learning efficacy while effectively managing computational expenses. Rapid convergence indicates that the model can efficiently grasp and assimilate critical features, swiftly reaching the desired performance level. This accelerates the feedback loop from data input to precise prediction.

The architecture of YOLOv5’s integrated detection head enables the sharing of multi-dimensional parameters across classification and localization tasks. This approach is designed to optimize the performance equilibrium between these two tasks synergistically. In the realm of road crack detection, the convolutional head (conv-head) and the fully connected head (fc-head) demonstrate distinct biases: the fc-head excels in crack type classification, whereas the conv-head is more adept at crack localization. It is imperative to acknowledge the indispensability of both heads for precise road crack detection. Further analysis revealed that the fc-head exhibits heightened sensitivity to spatial resolution compared to the conv-head. This grants the fc-head the capability to discern subtle distinctions between entire crack areas and their localized features. However, this characteristic also implies potential instability for the fc-head in global object localization regression tasks.

Hence, when crafting and refining the YOLOv5 model, meticulous attention must be paid to the attributes of both the classification and localization tasks, as well as their interplay. This ensures the attainment of an optimal equilibrium, wherein each task maintains peak performance while enabling the model to deliver highly efficient and precise prediction outcomes. This encompasses proficient identification of crack categories and accurate localization judgments.

In response to the challenges outlined above, Dai et al. (2021) introduced Dyhead, a dynamic object detection head structure designed to enhance the expressive capability of the detection head while circumventing the need for additional computational resources. However, when employed for the purpose of road crack detection, Dyhead, despite its innovation, was experimentally demonstrated to potentially diminish the model’s average precision (mAP) and recall. This constraint warrants careful consideration when integrating Dyhead with the YOLOv5 model and deploying it on edge computing devices.

Efficient Decoupled Head (EDH) is a design scheme for decoupled heads, which employs a fused-channel strategy to create a more efficient and separate detection head. This scheme delineates between localization and classification tasks, treating them as independent entities, and augments model performance through a decoupling mechanism. In the classification task processing, a fully connected layer (fc-head) is utilized to enhance classification accuracy and localization precision. The specific loss function is:

In traditional detection heads, classification and localization share a single convolutional kernel, represented as:


(1) $$y=W^{*}x$$

where: $C_{cls}\left(i,j\right)$ denotes the classification loss of the i -th prediction frame and the j -th true frame, y denotes the output feature map, W denotes the convolutional kernel, x denotes the input feature map.

In decoupled detection heads, the classification and localization tasks are handled by separate convolutional kernels:


(2) $$y_{cls}=W_{cls}{\;}^{*}x$$

(3) $$y_{reg}=W_{reg}{\;}^{*}x$$

where: $y_{cls}$ and $y_{reg}$ denotes the output feature maps for classification and localization, respectively, $W_{cls}$ and $W_{reg}$ denotes the convolutional kernels used for classification and localization, respectively.

The primary concept of the Efficient Decoupled Head (EDH) is to decouple the classification and regression tasks. Independent network heads are designed for each task. Assuming the input feature map is F , separate classification and regression heads are designed to handle the classification and localization tasks, respectively.


(4) $$P_{cls}=conv_{cls}\left(F\right)$$

where: $P_{cls}$ denotes the classification prediction results, $conv_{cls}$ denotes the convolution operation used for regression.

A joint loss function is used to simultaneously optimize the classification and regression tasks. The classification loss typically employs the focal loss, which is formulated as follows:


(5) $$L_{f}=-a_{t}{\left(1-p_{t}\right)}^{\gamma }\log\left(p_{t}\right)$$

where: $L_{f}$ denotes the focal loss. $p_{t}$ is the predicted probability of the model for the true class t , $a_{t}$ denotes the balancing factor, which is used to balance the ratio of positive to negative samples, γ is the focusing parameter, used to adjust the weights of easy-to-classify and hard-to-classify samples.

The regression loss employs the Smooth L1 Loss, defined as:


(6) $$L_{reg}=\frac{1}{N}\overset{N}{\underset{i=1}{\sum }}\overset{4}{\underset{j=1}{\sum }}Smooth_{L1}\left(t_{ij}-t_{ij}^{*}\right)$$

where: $L_{reg}$ denotes the regression loss, N denotes the number of samples, $t_{ij}$ denotes the predicted value of the j -th bounding box parameter for the i -th sample, $Smooth_{L1}$ denotes the Smooth L1 Loss function.

The total loss is the weighted sum of the classification loss and the regression loss:


(7) $$L=L_{cls}+\lambda L_{reg}$$

Where: λ is the weighting coefficient that balances the classification and regression losses. L denotes the total loss.

The model’s complexity was diminished by consolidating the 3 × 3 convolutional layers in the middle layer into a single layer, alongside adjusting the head’s width according to the width multipliers of the backbone and neck. Furthermore, this study employs an anchorless detector, which forecasts the distance from the anchor point to each edge of the object bounding box via a box regression branch, thus augmenting the model’s detection accuracy. These enhancements not only alleviate the computational load of the model but also bolster its efficacy in real-world scenarios.

### 3.2 Optimizing the loss function for bounding box regression

At present, YOLOv5 extensively employs the CIOU loss function. This function not only evaluates the overlap area between the predicted and actual bounding boxes but also introduces the centroid distance metric and considers differences in aspect ratio. As a result, it provides a comprehensive metric that aids in more accurate alignment of predicted and actual bounding boxes. Compared to the previous IOU loss function, the CIOU loss function demonstrates faster convergence and greater stability during the training process, as fully confirmed in experiments. The formula for deriving this function is:


(8) $$IOU=\frac{\left(A\cap B\right)}{\left(A\cup B\right)}$$

(9) $$L_{\mathrm{IoU}}=1-IOU$$

(10) $$v=\frac{4}{\pi^{2}}{\left(\arctan\frac{w^{gt}}{h^{gt}}-\arctan\frac{w^{prd}}{h^{prd}}\right)}^{2}$$

(11) $$\alpha =\frac{v}{\left(1-IOU\right)+v}$$

(12) $$L_{\mathrm{CIoU}}=1-\left(\mathrm{IoU}-\frac{\rho^{2}\left(b^{gt}{\mathrm{,b}}^{prd}\right)}{c^{2}}-\alpha v\right)$$

where: IOU denotes the cross-combination ratio, A and B represent the area of the true frame of the prediction frame, respectively, $L_{\mathrm{IoU}}$ denotes the IOU loss function, v is used to measure the consistency of the relative proportions of two rectangular boxes, $w^{prd}$ and $h^{prd}$ denote the width and height of the prediction box, respectively, $w^{gt}$ and $h^{gt}$ denote the width and height of the real box, respectively, α is the weighting factor, $L_{\mathrm{CIoU}}$ denotes the CIOU loss function, $b^{prd}$ denotes the center of the prediction box, $b^{gt}$ denotes the center point of the real frame, ρ denotes the Euclidean distance between two rectangular boxes, c denotes the distance between the diagonals of the closed regions of two rectangular boxes.

Although the CIOU loss function has made significant progress in object detection tasks, its computational complexity remains a challenge for edge computing devices, particularly during the training process of road crack detection models. The data collected from the external environment can be complex, leading to a large computational burden during the recognition process. Additionally, the CIOU loss function may cause the prediction box to unreasonably expand in certain cases, and reducing the loss value may not result in accurate detection, because the function prioritizes reducing the distance from the bounding box’s center point, disregarding the precision of the bounding box dimensions.

To address these limitations, this paper proposes the use of the MPDIOU loss function as an alternative to the CIOU loss function in YOLOv5. The MPDIOU loss function considers overlapping regions, centroid distances, and deviations in widths and heights, when evaluating the similarity between predicted and actual boxes. This method is well-suited for edge computing devices as it simplifies the comparison of similarities between bounding boxes. The MPDIOU loss function enhances computational efficiency in both overlapping and non-overlapping bounding box regression tasks, thereby improving the model’s accuracy in real-world scenarios.

MPDIOU aims to minimize the distance between the top-left and bottom-right points of the predicted box and the actual box. The formula for this derivation is as follows:.

Define the fixed point coordinates, and for the real bounding box $B_{gt}$ and the predicted bounding box $B_{prd}$ , define their vertex coordinates:

Any two convex shapes A , $B\subseteq S\in R^{n}$ , for A and B , $\left(x_{1}^{A},y_{1}^{A}\right)$ , $\left(x_{2}^{A},y_{2}^{A}\right)$ denote the coordinates of the upper left and lower right points of A . $\left(x_{1}^{B},y_{1}^{B}\right)$ , $\left(x_{2}^{B},y_{2}^{B}\right)$ denote the coordinates of the upper left and lower right points of B .

Calculate the Euclidean distance between the top left and bottom right points:


(13) $$d_{1}^{2}={\left(x_{1}^{B}-x_{1}^{A}\right)}^{2}+{\left(y_{1}^{B}-y_{1}^{A}\right)}^{2}$$

(14) $$d_{2}^{2}={\left(x_{2}^{B}-x_{2}^{A}\right)}^{2}+{\left(y_{2}^{B}-y_{2}^{A}\right)}^{2}$$

Based on the above distances, MPDIOU is calculated as:


(15) $$MPDIOU=\frac{A\cap B}{A\cup B}-\frac{d_{1}^{2}}{w^{2}+h^{2}}-\frac{d_{2}^{2}}{w^{2}+h^{2}}$$

Using MPDIOU as a loss function, it is defined as follows:


(16) $$L_{MPDIOU}=1-MPDIOU$$

The four-point coordinates can be used to determine all factors of the existing bounding box regression loss function. Use the following conversion formula:


(17) $$\begin{array}{l} \mathrm{|C|}=\left(\max\left(x_{2}^{gt},\;x_{2}^{prd}\right)-\min\left(x_{1}^{gt},\;x_{1}^{prd}\right)\right)\\ {}*\left(\max\left(y_{2}^{gt},\;y_{2}^{prd}\right)-\min\left(x_{1}^{gt},\;x_{1}^{prd}\right)\right) \end{array}$$

(18) $$x_{c}^{gt}=\frac{x_{1}^{gt}+x_{1}^{gt}}{2},y_{c}^{gt}=\frac{y_{1}^{gt}+y_{1}^{gt}}{2}$$

(19) $$x_{c}^{prd}=\frac{x_{1}^{prd}+x_{2}^{prd}}{2},y_{c}^{prd}=\frac{y_{1}^{prd}+y_{2}^{prd}}{2}$$

(20) $$w_{gt}=x_{2}^{gt}-x_{1}^{gt},h_{gt}=y_{2}^{gt}-y_{1}^{gt}$$

(21) $$w_{prd}=x_{2}^{prd}-x_{1}^{prd},h_{prd}=y_{2}^{prd}-y_{1}^{prd}$$

where $d_{1}^{2}$ and $d_{2}^{2}$ denote the square of the distance between the upper left and lower right points of A and B , $L_{\mathrm{MPDIoU}}$ denotes *MPDIOU* loss function, w and h denote the width and height of the input image, |C| denotes the smallest outer rectangle that covers both the real and predicted bounding boxes, ( $x_{c}^{gt}$ , $y_{c}^{gt}$ ) and ( $x_{c}^{prd}y_{c}^{prd}$ ) denote the coordinates of the center points of the real and predicted bounding boxes, respectively, $w_{gt}$ and $h_{gt}$ denote the width and height of the real bounding box, $w_{prd}$ and $h_{prd}$ denote the width and height of the predicted bounding box.

### 3.3 Introduction of the C3 module of the Global Context Block

Deploying YOLOv5 models in edge computing environments presents several challenges,. Because of the limited computing power and memory that edge devices typically have. The complexity of YOLOv5 is a test for resource-constrained edge devices. To address this issue, compression or pruning operations may be necessary. But these processes can negatively impact the model’s detection accuracy.

Furthermore, despite the faster detection speed of YOLOv5, computational power limitations on edge devices may still hinder their real-time object detection goals. Therefore, it is crucial to develop more efficient and lightweight object detection models that meet the specific needs of these devices. Such models should minimize their dependence on computational resources while maintaining high detection accuracy, thus meeting the accuracy requirements in edge computing environments.

Qiao et al. (2021) proposed the Switchable Atrous Convolution (SAconv) method to more accurately identify and segment objects in an image. This is achieved by applying different null convolution rates to the same input features for convolution. Additionally, a switching function is used to combine the results of the convolution with different null rates, making the network more flexible for feature size and scale. However, while the application of SAconv in road crack detection improves the model’s performance, it also consumes a significant amount of GPU resources thereby slowing down the model’s training speed, which is not conducive to deploying the YOLOv5 model on edge computing devices.

To address the issue mentioned above, the C3 module of YOLOv5 introduces the Global Context Block. This block performs global context modeling on the input feature graph to obtain global context information. GCBlock computes the pairwise relationship between the query location and all other locations to form an attention graph. The features of all locations are then weightedly aggregated with the attention graph. The aggregated features and the features of each query location are used to derive the output. Additionally, GCBlock captures inter-channel dependencies. GCBlock maps the weights in the attention graph to the channel dimensions of the feature graph. It then performs a feature transformation using a 1 × 1 convolution for inter-channel dependency transformation. Finally, GCBlock fuses the global context features with the inter-channel dependency transformed features to obtain the final output. The exact mathematical derivation is as follows:

Let the input feature map be $X\in R^{C\times H\times W}$ . Where C is the number of channels, H and W are the height and width of the feature map, respectively.

Global Average Pooling (GAP) is performed on the input feature map to obtain the global context features G :


(22) $$G=\frac{1}{H\times W}\overset{H}{\underset{i=1}{\sum }}\overset{W}{\underset{j=1}{\sum }}X_{ijk}$$

where $G\in R^{C}$ denotes the global average for each channel.

The global context feature G is transformed through a Fully Connected Layer (FCL) to obtain the transformed feature $\tilde{G}$ ;


(23) $$\tilde{G}=W_{g}G+b_{g}$$

where $W_{g}$ and $b_{g}$ are the weights and biases of the fully connected layer, respectively.

Next, the transformed global context feature $\tilde{G}$ is fused with the input feature map X through a channel attention mechanism:


(24) $$Y=X+\tilde{G}\cdot \sigma \left(W_{y}\tilde{G}+b_{y}\right)$$

Where: $W_{y}$ and $b_{y}$ are the parameters of the channel attention mechanism, σ is the activation function (e.g., Sigmoid function) $Y\in R^{C\times H\times W}$ is the output feature map.

The channel attention mechanism is used to weight different channels with the specific formula:


(25) $$A=\sigma \left(W_{a}\tilde{G}+b_{a}\right)$$

Where: $W_{a}$ and $b_{a}$ are the weights and biases of the channel attention mechanism, respectively, and $A\in R^{C}$ is the channel attention coefficient.

Finally, the channel attention coefficient A is applied to the input feature map X :


(26) Z=X⋅A

Where: $Z\in R^{C\times H\times W}$ is the weighted feature map.

By introducing Global Context Block, the C3 module can enhance the perception of global context information while preserving the original local features. The specific process is as follows:

1. The input feature map X is passed through multiple convolutional layers to obtain the intermediate feature map $X^{\prime }$ .
2. Input $X^{\prime }$ into the Global Context Block to get a feature map that incorporates the global context information Z .
3. Fuse Z with the input feature map X to get the final output feature map Y .

**Figure 2:**
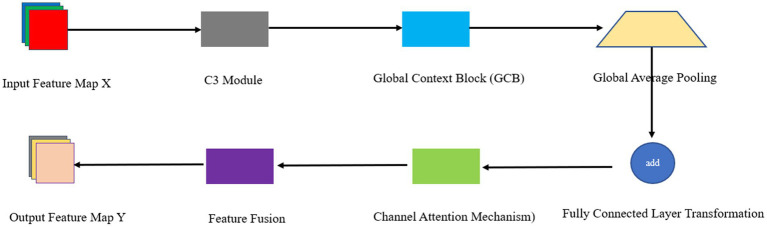
GCC3 structure.

The Backbone component is a crucial element in the YOLOv5 architecture, responsible for extracting features from the input data, particularly in the shallow part of the network. However, capturing shallow features becomes increasingly challenging as the network’s depth increases. For this reason, the feature extraction process can be effectively enhanced through global modeling relationships. The Global Context Block enhances the network’s ability to capture distant correlations in the image through expanding the existing sensory field, which in turn improves the understanding of the object’s contextual information. Combined with the C3 structure, this approach extends the receptive field at different levels and enhances the global perception capability of the network. The C3 structure builds a feature pyramid network to generate multi-scale feature maps. When combined with the Global Context Block, global context information can be introduced at all scales, thus significantly enhancing the feature representation. Based on this, we propose the GCC3 module to replace the traditional C3 module in YOLOv5. This will optimize the feature extraction process and improve the overall performance of the model. The network architecture for this module is shown in Figure 2.

### 3.4 YOLOv5 model improvements

**Figure 3:**
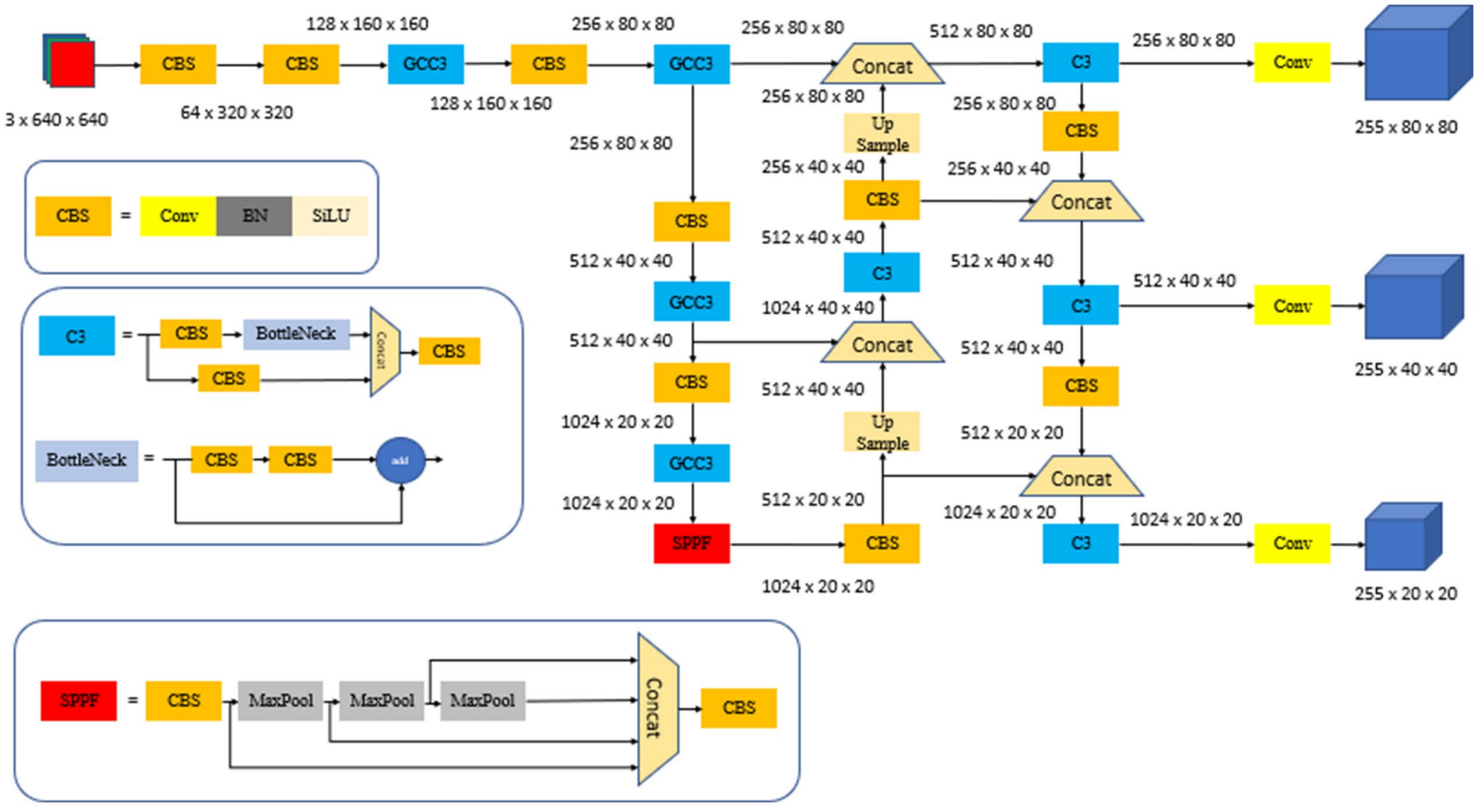
EMG-YOLO structure.

This study proposes an enhanced YOLO-EMG detection algorithm to alleviate the performance limitations encountered by YOLOv5, when deployed in edge computing environments. The algorithm effectively resolves the conflict between classification and localization tasks within the model by introducing an efficient decoupled head structure. This leads to a significant reduction in the model’s reliance on computational resources and expedites training convergence. Additionally, optimization of YOLOv5’s CIOU loss function is achieved by implementing a more efficient MPDIOU loss function. This not only decreases computational overhead during training but also addresses the potential issue of the CIOU loss function amplifying prediction frame errors while simultaneously reducing loss values. By integrating the GCC3 module in place of the traditional convolutional layer, the EMG-YOLO algorithm enhances real-time detection performance on edge computing devices, while preserving model accuracy and precision. The EMG-YOLO architecture is depicted in Figure 3.

## 4 Results

### 4.1 The data set and the experimental environment

To demonstrate the efficiency of the proposed YOLO-EMG for road crack detection on edge computing devices, this paper utilizes two datasets: the RDD2022 dataset, which contains over 20,000 new photos compared to RDD2020 and covers six countries (Japan, India, Czech Republic, Norway, USA, and China), and a dataset on road damage. Although this dataset of 47,420 images of road damage cannot be directly used in the YOLO algorithm, it can be made suitable for the algorithm through cleaning and format conversion processing of the data. The other dataset is from the CrackForest dataset, which gives a general picture of urban pavement conditions. This dataset is mainly used for the image recognition task of automatic crack and damage detection. The four road distresses in the dataset. The meaning of each category is shown in Table 1.

**Table 1:** Meaning of various crack labels.

Label name	Crack name
D00	Longitudinal cracks
D10	Transverse cracks
D20	Meshlike cracking
D40	Pavement pothole

The experiment was conducted on a Windows 10 operating system, using an NVIDIA GeForce RTX2080Ti GPU with 8 GB of RAM. The software environment included CUDA 11.3 and Python 3.10. The experimental code was based on YOLOv5-master, with the initial learning rate set to 0.01, the batch size set to 8, and the input image resolution set to 640 × 640. The experiment was run for 50 epochs, with all other parameters set to their default values. The performance metrics include mean average precision (mAP) which reflects the object localization effect and bounding box regression capability. It is calculated using IOU thresholds ranging from 0.5 to 0.95. Additionally, the mean accuracy (mAP) is calculated using IOU thresholds of 0.5 and 0.5: 0. The model’s performance is evaluated based on its objectivity, comprehensibility, logical structure, conventional structure, clear and objective language, adherence to formatting guidelines, formal register, balanced approach, precise word choice, and grammatical correctness. The evaluation metrics include model accuracy (mAP), model size (M), volume (MB), GFLOPS (G), and frames per second (FPS). The mAP (0.5) reflects the mean accuracy when the IOU threshold is 0.5, which mainly indicates the recognition ability of the object detection model.

### 4.2 Ablation experiment results

To verify the effectiveness of the introduced modules, their performance was hypothesized and subsequently validated through ablation experiments Efficient Decoupled Head: The Efficient Decoupled Head was introduced to separate the classification and localization tasks. It is hypothesized that this separation can improve the model’s training efficiency and detection accuracy. MPDIOU Loss Function: The MPDIOU loss function was introduced to account for overlapping regions, central point distances, and width and height discrepancies, thereby reducing bias during the training process. It is hypothesized that this optimization can enhance the model’s computational efficiency on edge devices. GCC3 Module: The Global Context Block was introduced into the C3 module, with the hypothesis that this module can enhance feature extraction capabilities through global contextual information, thereby improving the model’s performance in complex environments.

To validate the effect of each enhancement module of EMG-YOLO on the whole model, this experiment sequentially adds each module to the original YOLOv5 model. Ablation experiments are then performed on two road crack datasets to validate the effectiveness of the present model. The three enhancements tested are denoted by the acronyms E (Efficient Decoupling Header), M (MPDIOU) and G (GCC3), with a tick indicating the use of the module. The results are displayed in Table 2.

**Table 2:** Results of EMG-YOLO ablation experiments.

E	M	G	Image size	mAP (0.5:0.95)	mAP (0.5)	Precision	Volume/MB	GFLOPS (G)
			640 × 640	0.252	0.489	0.554	14.60	16.00
**√**			640 × 640	0.254	0.495	0.558	17.11	18.75
	**√**		640 × 640	0.257	0.497	0.560	17.22	20.23
		**√**	640 × 640	0.256	0.491	0.555	19.95	21.87
**√**	**√**		640 × 640	0.259	0.507	0.559	23.45	25.71
	**√**	**√**	640 × 640	0.260	0.512	0.563	28.14	28.95
**√**		**√**	640 × 640	0.259	0.509	0.561	26.42	27.64
**√**	**√**	**√**	640 × 640	0.261	0.515	0.569	28.20	29.60

Analysis of experimental results: The Efficient Decoupled Head (E) enables each task to optimize its respective features independently by separating the classification and localization tasks. This design reduces interference between tasks, especially when dealing with complex scenarios. The results show that the mAP (0.5, 0.95) of the model improves from 0.252 to 0.254 and the mAP (0.5) improves from 0.489 to 0.495 with the use of the Efficient Decoupling Header. this indicates that the separation of the classification and localization tasks effectively improves the overall performance of the model.

The MPDIOU loss function (M) optimizes bounding box regression by more accurately calculating the overlap region and distance between the predicted and real boxes. Compared with the traditional IOU loss function, MPDIOU takes more geometric information into account. Thus, it reduces the bias in the training process. The experimental results show that the mAP (0.5:0.95) of the model improves from 0.252 to 0.257 and the mAP (0.5) improves from 0.489 to 0.497 with the use of MPDIOU. indicating that the accuracy of the bounding box localization is significantly improved.

The GCC3 module (G) enhances the feature extraction by introducing global context information. Compared with the traditional convolutional layer, GCC3 is able to better capture the relationship between global and local features, thus improving the detection performance of the model. The results show that the mAP (0.5:0.95) of the model improves from 0.252 to 0.256 and the mAP (0.5) improves from 0.489 to 0.491 with the use of GCC3, which proves the importance of global contextual information in feature extraction.

By combining the E and M modules, the model performs well in optimizing the localization and classification tasks: the Efficient Decoupled Head reduces the interference between the classification and localization tasks, allowing the model to better focus on their respective tasks; the MPDIOU loss function further improves the accuracy of the boundary regression by taking into account more geometric information, making the prediction of position and dimensions more accurate. This combination significantly improves the detection performance and accuracy of the model.

The model’s edge regression accuracy and feature extraction capability are enhanced by combining the M and G modules: the MPDIOU loss function improves the accuracy of edge regression by taking geometric information into account, and the GCC3 module improves the detection performance by allowing the model to better understand and extract feature information in the image through the introduction of global contextual information. This combination performs particularly well in complex environments, enhancing the model’s detection accuracy and reliability in complex scenes.

The feature extraction capability and task-independent optimization of the model are enhanced by combining the E and G modules. The Efficient Decoupled Head reduces inter-task interference, allowing the model to better focus on their respective tasks; the GCC3 module enhances the feature extraction capability by the introduction of global contextual information. This combination enables the model to better identify and locate targets in complex contexts, improving detection accuracy and reliability.

The above analysis shows that each enhancement module of EMG-YOLO has a positive impact on the model performance. They can significantly improve the detection accuracy and efficiency of the model when used in combination. These improvements make the application of EMG-YOLO in edge computing environment more effective and reliable.

### 4.3 Mainstream algorithm comparison experiment

To verify the efficiency and effectiveness of this model, an experimental comparison is made between EMG-YOLO and mainstream algorithms under the same experimental conditions, and the selected comparison models are mainly the following: the two-stage object detection models with high detection accuracy, Faster R-CNN and SSD, EfficientDet, CentreNet (Resnet50). The above models are trained and tested in the same environment, and the model performance is comprehensively compared using metrics such as mean accuracy (mAP), number of parameters and detection frame rate (FPS). Table 3 shows that the two-stage object detection model Faster R-CNN has lower accuracy than most one-stage object detection models, as well as more model parameters and lower detection efficiency. Additionally, SSD has lower detection accuracy and performs poorly in small object detection scenarios. The results are displayed in Table 3.

**Table 3:** Comparison of detection results between EMG-YOLO and other five methods.

Model	Image size	mAP (0.5:0.95)	mAP (0.5)	Precision	Volume/MB	GFLOPS (G)
Faster R-CNN	640 × 640	0.258	0.450	0.493	108.3	275.6
SSD	640 × 640	0.203	0.401	0.439	92.1	217
EfficientDet	640 × 640	0.200	0.396	0.434	25.7	6.2
CenterNet (Resnet50)	640 × 640	0.215	0.451	0.494	124.9	108
YOLOv5	640 × 640	0.252	0.489	0.554	14.6	16
EMG-YOLO	640 × 640	0.261	0.515	0.569	28.2	29.6

The experimental results show that the two-stage object detection model Faster R-CNN not only has lower accuracy than most one-stage object detection models, but also has more model parameters and lower detection efficiency. SSD has lower detection accuracy and performs poorly in small object detection scenarios in the crack dataset. EfficientDet is optimized in terms of model size and computational effort. But its detection accuracy is relatively lower. Especially when dealing with high resolution images and complex scenes. It does not perform as well as the YOLO series of models. CenterNet (Resnet50) achieves object detection through keypoint detection. Although it performs well in some scenes. However, its overall detection accuracy and efficiency are still not comparable to YOLOv5 and EMG-YOLO. YOLOv5 performs well in the single-stage object detection model. It strikes a good balance between detection accuracy and speed. However, EMG-YOLO further improves its performance through a variety of optimization measures. EMG-YOLO shows a clear advantage in detection accuracy over YOLOv5, with mAP (0.5) and mAP (0.5:0.95) improving by 2.9 and 0.9% respectively, and accuracy improving by 2.7%.

Comparative analysis of related models shows that Faster R-CNN, as a two-stage detection model, requires considerable computational resources for region proposal and classification steps. This makes it inefficient on edge computing devices. Additionally, its large number of parameters results in poor performance in scenarios that demand high processing speed and real-time response. In road crack detection, rapid response and efficient computation are critical, which Faster R-CNN struggles to meet.

EMG-YOLO addresses these issues by incorporating an Efficient Decoupled Head and GCC3 module, significantly reducing computational resource requirements and enhancing detection speed, making it particularly suitable for edge computing devices. Moreover, the MPDIOU loss function improves detection accuracy, solving the problem of accuracy degradation on edge devices that Faster R-CNN faces.

While SSD models are faster, their performance in detecting small objects is suboptimal. Road cracks are typically small and complex, and SSD’s detection accuracy is insufficient to effectively identify these fine cracks, resulting in overall poor detection performance.

EMG-YOLO significantly enhances the model’s ability to detect small objects and improve accuracy. By reducing interference between classification and localization tasks and optimizing feature map utilization, the training computational burden is lessened. Additionally, EMG-YOLO’s enhanced feature extraction capability ensures high-accuracy detection even in complex environments, addressing SSD’s shortcomings.

EfficientDet has optimized model size and computational efficiency, yet its detection accuracy in high-resolution images and complex scenarios remains inadequate. Road crack detection demands high-precision feature extraction and classification, where EfficientDet’s capabilities are relatively limited.

EMG-YOLO, through global context modeling, strengthens feature extraction capabilities, maintaining high-accuracy detection in complex environments. Compared to EfficientDet, EMG-YOLO demonstrates better adaptability and performance on edge computing devices.

CenterNet (Resnet50) employs keypoint detection for object detection and performs well in certain scenarios. However, its effectiveness in detecting complex and diverse cracks is limited. CenterNet’s balance between processing speed and accuracy is inferior to YOLOv5 and EMG-YOLO, resulting in less satisfactory performance in practical applications.

YOLOv5 excels in single-stage object detection with good detection speed and accuracy. However, EMG-YOLO further optimizes feature extraction and localization precision by integrating an Efficient Decoupled Head (E), MPDIOU loss function (M), and GCC3 module (G), making it superior in road crack detection. The Efficient Decoupled Head reduces interference between classification and localization tasks, the MPDIOU loss function improves bounding box regression accuracy, and the GCC3 module enhances global context information in feature extraction. These improvements collectively elevate EMG-YOLO’s detection performance.

### 4.4 Comparative algorithmic experiments in edge computing devices

To tangibly demonstrate the model’s improved performance on edge computing devices, we deploy it on the Jetson Xavier NX edge device and compare its performance to that of the YOLOv5 model. The foundational parameters of the edge computing devices are detailed in Table 4.

**Table 4:** List of basic parameters of edge computing devices.

Jetson Xavier NX technical parameters			
AI performances	21TOPS	Vision accelerator	7 way VLIW vision processor
GPU	384-core NVDIA Volta GPU and 48Tensor cores	Camera	MIPI CSI-2 × 2(15bit Flex connector)
CPU	6-core NVIDIA Carmel ARMv8.2 64-bit CPU	Video decoding	2 × 4kp30|6 × 1080p60|14 × 1080p60|32 × 1080p30
RAM	8G128-bit LPDDR4x 51.2GB/S	Display	2multi-mode DP 1.4/eDP 1.4/HDMI 2.0

Due to the limited resources of edge computing devices, model performance may be affected. Both YOLOv5 and EMG-YOLO have been optimized for edge computing, making them more suitable for comparison in such environments. Other more complex or unoptimized models may not run efficiently on edge computing devices, rendering experimental results less meaningful. Among the models compared, YOLOv5 performs optimally. EMG-YOLO is an improved model based on YOLOv5, and directly comparing these two models can more clearly demonstrate the effectiveness of the improvements. Introducing other models would complicate the comparison results, making it difficult to highlight the advantages of EMG-YOLO over YOLOv5. By maintaining consistent experimental conditions and comparing only these two models, variable control is improved, ensuring the reliability and consistency of the experimental results. This approach helps avoid additional variable interference caused by model complexity or other factors, thereby ensuring the accuracy of the experimental conclusions. The comparison chart of specific experimental results is shown below.

**Figure 4:**
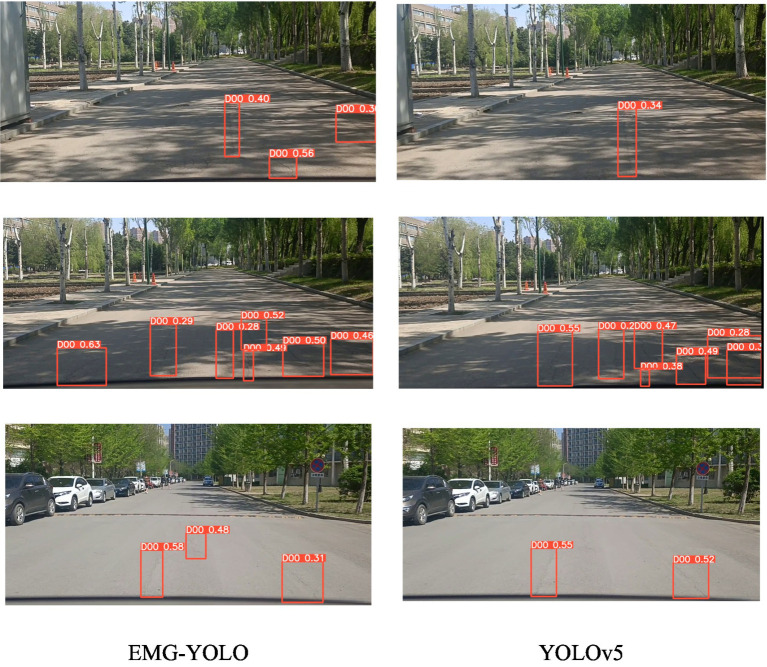
Comparison results in shaded environments.

Visualization Results Analysis:In shadow, foggy, and nighttime environments, the extraction of features becomes challenging due to complex backgrounds and varying lighting conditions. The Efficient Decoupled Head separates classification and localization tasks, allowing each to focus on its specific features, thereby reducing information confusion and task interference. In shadowy environments (Figure 4), EMG-YOLO is able to more accurately identify crack edges and shapes, whereas YOLOv5 exhibits noticeable omissions and false detections under similar conditions. This validates the effectiveness of the Efficient Decoupled Head in complex environments, enhancing the model’s robustness and detection accuracy.

**Figure 5:**
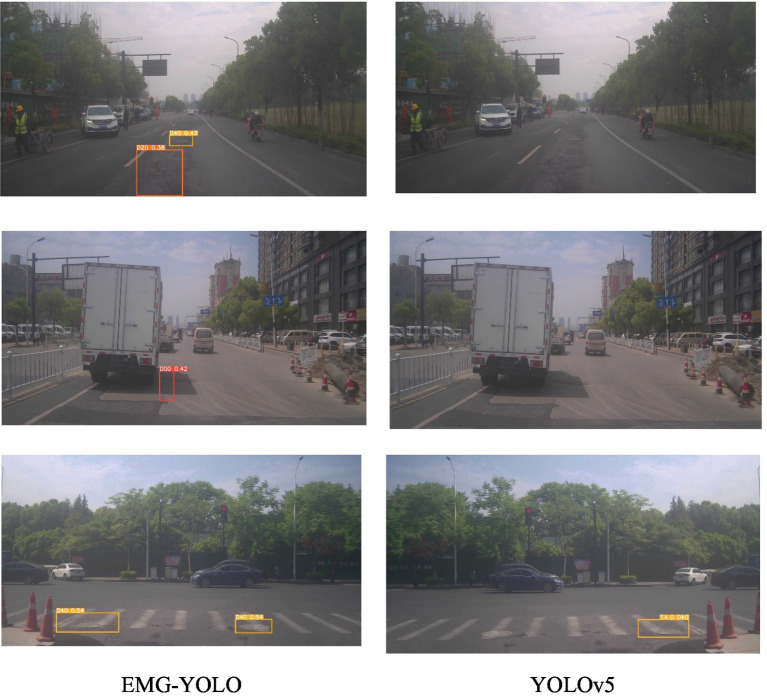
Comparison effect in foggy environment.

The MPDIOU loss function optimizes bounding box regression by considering geometric information of the bounding box. In foggy environments (Figure 5), reduced visibility makes bounding box localization more difficult. EMG-YOLO maintains high detection accuracy under these conditions, accurately regressing the position and size of cracks. This demonstrates that the MPDIOU loss function can provide more accurate regression results when handling highly overlapping and geometrically diverse bounding boxes, reducing the likelihood of missed and false detections, thus improving the model’s accuracy.

**Figure 6:**
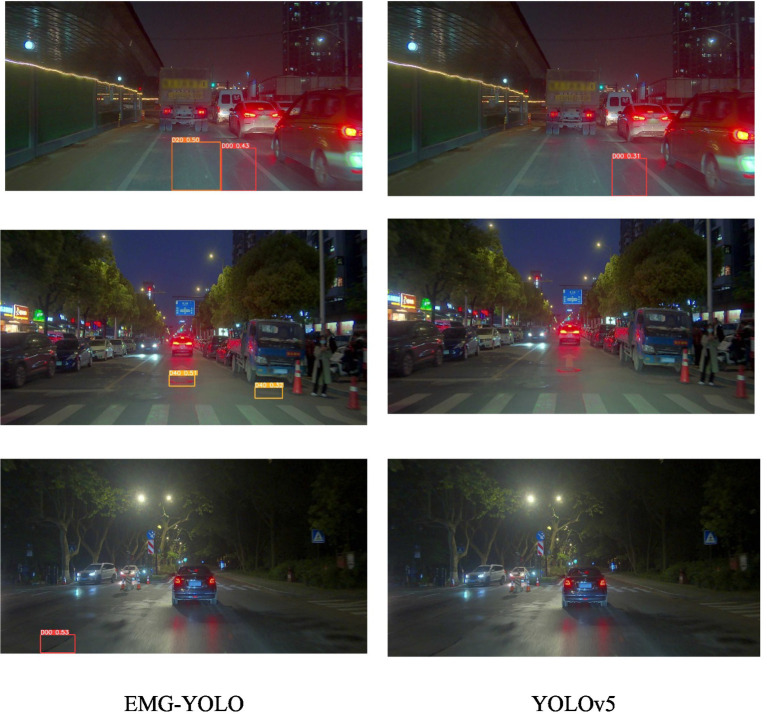
Comparison effect in night environment.

The GCC3 module enhances feature extraction capabilities by incorporating global contextual information. In nighttime environments (Figure 6), background noise and low light conditions increase the difficulty of feature extraction. Through the GCC3 module, EMG-YOLO better captures the relationship between global and local features, identifying crack features in complex backgrounds. In contrast, YOLOv5’s detection performance is significantly poorer under low light conditions. This indicates that the GCC3 module provides stronger feature representation capabilities in complex environments, enabling the model to detect cracks more accurately.

## 5 Conclusion

In this article, a road crack detection algorithm EMG-YOLO is proposed. It aims to solve the problem of data quality degradation caused by the direct connection of edge devices to the sensors, as well as the additional computational pressure on the model caused by the noise interference, which in turn results in the degradation of the model’s accuracy. The algorithm makes a series of improvements on the infrastructure of YOLOv5, including the integration of the GCC3 module to enhance the feature extraction capability, the adoption of MPDIOU instead of the traditional IOU loss function to improve the positioning accuracy, and the introduction of the Efficient decoupling header to optimize the network structure. These improvements enable the deployment of EMG-YOLO on edge computing devices not only to improve the accuracy of real time detection, but also to reduce the demand for computing resources. This means that in real world applications, EMG-YOLO can operate efficiently and provide reliable detection results, whether it is an inspection task on city roads, rural highways or remote areas. Therefore, the superior performance of EMG-YOLO on edge computing devices makes it a competitive solution for the current road crack detection task. Meanwhile, there are still some shortcomings in this paper, which need to be followed up with further research, the model does not take into account the robustness of the model for complex environments, and there is a leakage problem for the model for complex environments such as darkness, fog, etc., which will be a problem to be solved in the future.

## Data availability statement

The original contributions presented in the study are included in the article/supplementary material, further inquiries can be directed to the corresponding author.

## Author contributions

YX: Writing – review & editing. XH: Writing – original draft. XP: Validation, Writing – review & editing. DA: Writing – review & editing. WL: Validation, Writing – review & editing. YB: Resources, Writing – review & editing.
